# Cardiopulmonary bypass has a modest association with cancer progression: a retrospective cohort study

**DOI:** 10.1186/1471-2407-13-519

**Published:** 2013-11-03

**Authors:** Cathy Anne Pinto, Stephen Marcella, David A August, Bart Holland, John B Kostis, Kitaw Demissie

**Affiliations:** 1 Department of Epidemiology, Rutgers, Piscataway, NJ, USA; 2 Division of Surgery Oncology, Department of Surgery, The Rutgers Cancer Institute of New Jersey and Robert Wood Johnson Medical School, New Brunswick, NJ, USA; 3 Department of Preventive Medicine, Rutgers New Jersey Medical School, Newark, NJ, USA; 4 Cardiovascular Institute, Rutgers-Robert Wood Johnson Medical School for the MIDAS Study Group, New Brunswick, NJ, USA; 5 The Rutgers Cancer Institute of New Jersey and Robert Wood Johnson Medical School, New Brunswick, NJ, USA

**Keywords:** Cardiopulmonary bypass, Cancer progression, Population-based cohort study

## Abstract

**Background:**

Given their frequency of occurrence in the United States, cancer and heart disease often coexist. For patients requiring open-heart surgery, this raises concern that the use of cardiopulmonary bypass (CPB) may cause a transient immunosuppression with the potential to promote the spread and growth of coexisting cancer cells. This study examined the association of cardiopulmonary bypass with cancer progression in a large population-based setting using linked data from several state-wide registries.

**Methods:**

A retrospective cohort study of cancer risk, stage, and mortality in 43,347 patients who underwent coronary artery bypass graft (CABG) surgery with and without CPB in New Jersey between 1998–2004 was conducted. A competing risk analogue of the Cox proportional hazards model with propensity score adjustment and regression on the cause-specific hazard was used to compute relative risk ratios (95% confidence intervals [CIs]) for patients undergoing CABG surgery with and without CPB.

**Results:**

An increased risk for overall cancer incidence (17%) and cancer-specific mortality (16% overall, 12% case fatality) was observed; yet these results did not reach statistical significance. Of 11 tumor-specific analyses, an increased risk of skin melanoma (1.66 [95% CI, 1.08-2.55: p=0.02]) and lung cancer (1.36 [95% CI, 1.02-1.81: p=0.03]) was observed for patients with pump versus off-pump open-heart surgery. No association was found with cancer stage.

**Conclusions:**

These results suggest that there may be a relationship between CPB and cancer progression. However, if real, the effect is likely modest at most. Further research may still be warranted with particular focus on skin melanoma and lung cancer which had the strongest association with CPB.

## Background

Cardiac disease and cancer occur commonly in the United States, and it is therefore not infrequent that patients who undergo open heart surgery also develop cancer. Cancer therapy generally should be performed as soon as possible after diagnosis, except in cases where surgery may take priority for patients who are at high risk of suffering a more imminent major cardiac event. Open-heart surgery with cardiopulmonary bypass (CPB) is known to cause a transient immunosuppression, as evidenced by increases in immunoregulatory factors including IL-10, a major immunoregulatory cytokine with inhibitory effects of IFN-γ, tumor necrosis factor, IL-1, IL-6, and IL-8. CPB has also been shown to increase TGF-β, a cytokine with several potent immunosuppressive and immunomodulatory effects that may contribute to negative feedback regulation of T cell-mediated immune response [1,2]. It is therefore possible that such biochemical changes may lead to clinically relevant changes in immune system function and cancer surveillance with the potential to promote the spread and growth of co-existing cancer cells [1,2]. Although changes in immunoregulatory factors caused by CPB are short-lived and not likely to induce carcinogenesis, it is plausible that CPB may be linked to cancer progression. Further research is warranted, and may provide insight into the optimal strategy for management of cancer patients with cardiovascular co-morbidities.

Few studies have examined the association between CPB and cancer progression. A recent posthoc analysis of 611 patients by Vieira et. al. examined the association of cardiac and non-cardiac mortality with coronary artery bypass surgery with CPB and non-surgical interventions (PCI, medical treatment) [3]. Compared with the non-surgical controls, CPB surgery was associated with a lower incidence of cardiac death (76.8% and 47.1%, respectively) and higher incidence of non-cardiac mortality, with a higher tendency toward cancer related death (7.2% and 20.6%, respectively). Two other recent studies examined the effects of CPB after cancer diagnosis on all-cause and cancer-specific mortality rates [4,5]. The results demonstrated that all-cause and cancer-specific mortality after CPB increased with shorter time intervals between diagnosis and the surgical intervention, especially for those patients with less than 2 years between the cancer diagnosis and subsequent cardiac procedure (p<0.0001).

Although data suggests an association between CPB and cancer progression, these results have not been consistently demonstrated. A study by Platell and colleagues in 33 patients with histologically proven colorectal showed a lower 5-year colon cancer-specific survival rate for those who underwent CPB surgery than for non-surgical controls: 34% and. 71%, respectively, p<0.05; HR=2.9 (95% CI: 1.5-4.4). However, a sensitivity analysis that excluded patients with Stage IV cancer (1 CPB patient, 1 control) showed no significant difference in the cancer-specific survival rate (p=0.1) [6]. Additionally, in a recent multicenter study of CPB and cancer progression performed by Suzuki et. al. in 74 patients with metastatic cancer who underwent open-heart surgery with and without CPB, no significant difference was observed in cancer-specific mortality (26.7% and 24.1%, respectively, p=0.8) [7].

Further research is warranted to enable a robust assessment of the association between CPB and cancer progression. The aim of the present study is to evaluate cancer incidence, stage, and cancer-specific mortality in a large population-based cohort who previously underwent open heart surgery with and without CPB using linked data from several state-wide registries.

## Methods

### Population and data source

The study population included patients who underwent at least one open-heart coronary artery bypass surgery (CABG), with no incidental valve surgery, who had a hospital discharge date between January 1, 1998 and December 31, 2004 in the state of New Jersey. Patients had no prior cancer diagnosis with cancer records dating back to 1979. Patients with open-heart valve surgery or CABG surgery with incidental valve surgery were excluded from the study as these patients have very different propensities for CPB compared to those with isolated CABG surgeries.

For those with isolated CABG surgery, probabilistic record linkage was used to match records in several state-wide registries including 1) the Myocardial Data Acquisition System (MIDAS), which includes hospital discharge records for patients with a myocardial infarction and other invasive cardiovascular procedures who have been admitted to New Jersey non-federal acute care hospitals [8], 2) the New Jersey Department of Health and Senior Services Open-Heart Surgery Registry (OHSR), which includes open-heart surgery data required to be reported by NJ cardiac surgery hospitals necessary to maintain licensing [9], and 3) the New Jersey State Cancer Registry (NJSCR), which is a population-based National Cancer Institute’s Surveillance, Epidemiology and End Results (SEER) Registry and North American Association of Central Cancer Registry (NAACCR) and collects data on all cancers diagnosed and/or treated in New Jersey [10].

Institutional review board approvals for the study were obtained from the Rutgers and the Department of Health and Senior Services. NJSCR data were publically available for research purposes and access to the data was granted by NJ State Cancer Epidemiology Services. Access and permission to use the linked MIDAS and NJ Open Heart Surgery Registry data were provided by the Cardiovascular Institute at Robert Wood Johnson Medical School. After data linkage was complete, data were combined to form a comprehensive database for analysis, and all personal identifying information needed for linkage of the registries were deleted from the source records.

### Endpoints

The primary endpoint was cancer-specific mortality. Cancer-specific case fatality for those with cancers diagnosed within 1-year, 2-years, and 4-years of surgery was also examined, as well as cardiovascular- and other cause-specific mortality. The main source of vital statistics for the study was MIDAS, which is linked annually with mortality data obtained from the National Center for Health Statistics. An additional sensitivity analysis of cancer-specific mortality was also performed using vital statistics data from the National Center for Health Statistics Multiple Cause of Death File, which lists as many as 20 contributing causes of death in addition to the reported underlying cause of death [11].

Other key secondary endpoints included cancer incidence and stage. Overall cancer incidence was examined and incidence for a subset of 11 commonly occurring cancers, including those thought to be more susceptible to chronic immune modulation (i.e. skin melanoma, non-Hodgkin lymphomas, kidney cancer).

### Statistical methods

Given the observational nature of the study, propensity scores were used as an adjustment in the model to help minimize bias related to differences in baseline risk factors, as those with and without CPB may differ in important prognostic factors related to outcome [12]. The propensity score model was developed using a stepwise logistic regression with CPB as the outcome variable in the model. The model was developed with baseline characteristics reported at the time of the first open-heart surgery. Univariate modeling was performed to identify potential confounders and covariates with a significant association with the outcome of mortality [13,14]. The discriminatory power of the model was assessed using the area under the receiver operating characteristic curve (AUC), or C statistic; however, this model diagnostic was not used to guide variable selection into the propensity score model. Asymmetric restriction of the propensity score distribution (‘trimming’) was applied to improve overlap of the propensity score distribution and improve baseline covariate balance between the groups.

For cancer-specific mortality and cancer incidence, the primary analysis was performed using a competing risk analogue of the Cox Proportional Hazard models [15]. To account for the correlation among patients within a hospital or surgeon cluster, a robust covariance matrix was used to compute hazard ratios and 95% confidence intervals as measures of relative risk [16]. The zero time for the analysis was the date of the first surgery, and patients were followed until the date of the event (death, cancer incidence) or December 31, 2006 (date of censorship), whichever came first. The proportional hazards assumption was tested using Schoenfeld residuals [17]. Each model was adjusted, as applicable, for potential confounders including age, gender, race, cancer type, stage, time interval between surgery and cancer diagnosis, type of cancer treatment, use of blood products during surgery, year of surgery, and propensity score quantiles. For each analysis, crude and adjusted hazard ratios are presented. Differences in cancer stage was examined using a Cochran Armitage trend test.

Logistic and linear regression models, diagnostic testing, and sensitivity analyses were also employed as appropriate. Given the exploratory nature of this research, no multiplicity adjustments were performed. All analyses were performed in SAS V9.2 (SAS Institute Inc, Cary, NC, USA).

## Results

### Sample and baseline covariates

A total of 48,009 patients who underwent isolated open-heart CABG surgery with (35,795) and without (12,214) CPB with extracorporeal circulation (or “pump” procedures) were included in the final analysis. Less than 2% of patients in the linked database were excluded from the analysis due to missing perfusion data or death on the date of open-heart surgery. The majority of patients (>99.7%) included in the final analysis had a single open-heart surgery and the mean perfusion time for surgeries performed on pump was 86 minutes. Additional baseline characteristics are summarized in Table 1.

**Table 1:** **Baseline characteristics for patients with open**-**heart surgery in New Jersey between 1998**–**2004**, **by CPB status**

		**All CABG patients**^**a **^**(n=48,009)**			
		**Pump**^**b **^**(n=35,795)**		**Off pump (n=12,214)**	
**Length of hospital stay**, mean (range)		11	(0–303)	10	(0–175)
**Males**, n (%)		25,868	(72.7)	8,547	(70.0)
**Age**, n (%)	<50	3,231	(9.0)	1,096	(9.0)
	50-59	7,439	(20.8)	2,351	(19.3)
	60-69	11,304	(31.6)	3,637	(29.8)
	70-79	10,999	(30.7)	3,743	(30.7)
	≥80	2,822	(7.9)	1,387	(11.4)
**Race/ethnicity**, n (%)	White	30,030	(83.9)	9,744	(79.8)
	Black	1,927	(5.4)	931	(7.6)
	Hispanic	1,511	(4.2)	757	(6.2)
	Other/unknown	2,327	(6.5)	782	(6.4)
**Primary insurance**, n (%)	Medicare/medicaid	16,254	(45.4)	6,450	(52.8)
	Blue cross/commercial	6,021	(17.3)	2,070	(17.0)
	HMO	7,801	(21.8)	2,351	(19.3)
	Uninsured/indigent	924	(2.6)	366	(3.0)
	Self pay	676	(1.9)	205	(1.7)
	Other	3,939	(11.0)	772	(6.3)
**Preoperative status**^**c**^, n (%)	Elective	13,428	(37.5)	4,297	(35.2)
	Urgent	20,369	(56.9)	7,412	(60.7)
	Emergent	1,793	(5.0)	403	(3.3)
	Salvage	78	(0.2)	13	(0.1)
**Myocardial infarction**, n (%)		15,934	(44.5)	5,073	(41.5)
**Prior CV intervention**, n (%)		8,987	(25.1)	2,823	(23.1)
**Diabetes**, n (%)		13,069	(36.5)	4,009	(32.8)
**Hypertension**, n (%)		27,153	(75.9)	8,779	(71.9)
**Congestive heart failure**, n (%)		5,981	(16.7)	2,034	(16.7)
**Cardiogenic shock**, n (%)		1,497	(4.2)	253	(2.1)
**Endocarditis**, n (%)		77	(0.2)	19	(0.2)
**Cerebrovascular disease**, n (%)		4,042	(11.3)	1,417	(11.6)
**Smoke**, **ever**, n (%)		18,724	(52.3)	6,454	(52.8)
**Renal failure**, n (%)		1,660	(4.6)	691	(5.7)
**Lung disease**, n (%)	Mild	3,427	(9.6)	1,078	(8.8)
	Moderate	753	(2.1)	294	(2.4)
	Severe	343	(1.0)	181	(1.5)
**Peripheral vascular disease**, n (%)		5,359	(15.0)	2,019	(16.5)
**Disease vessels**, n (%)	One	958	(2.7)	759	(6.2)
	Two	5,721	(16.0)	2,010	(16.5)
	Three	23,407	(65.4)	7,699	(63.0)
**LM disease** (>50% Occlusion), n (%)		10,055	(28.1)	3,208	(26.3)
**Ejection fraction** (%), n (%)	<20%	881	(2.5)	490	(4.0)
	20-29%	2,494	(7.0)	725	(6.0)
	30-39%	5,002	(14.0)	1,422	(11.7)
	40-49%	8,703	(24.3)	2,641	(21.6)
	≥50%	18,577	(51.9)	6,963	(56.8)

^a^includes patients with ≥1 isolated CABG surgeries; excludes patients with incidental valve surgery .

^b^includes patients with ≥1 on-pump procedure prior to incident cancer. Less than 0.2% of patients also had an off-pump procedure.

^c^elective: procedure deferred without increased risk of compromised cardiac outcome; urgent: not elective/emergent, required during same hospitalization to minimize chance of further clinical deterioration, worsening/sudden chest pain, congestive heart failure, acute myocardial infarction (AMI), anatomy, IABP, unstable angina with IV nitroglycerin or rest angina; emergent: ischemic dysfunction (ongoing ischemia including rest angina despite maximal medical therapy (medical and/or IABP), MI within 24 hours before surgery, or pulmonary edema requiring intubation), mechanical dysfunction (shock with or without circulatory support); emergent salvage: CPR enrout to the operating room or prior to anesthesia induction.

Baseline characteristics were assessed at the time of the first open-heart surgery.

%= n/N patients where n=frequency count in each category and N=total number of subjects.

Due to rounding, sum of all percentages may equal 100%.

CABG= coronary artery bypass graft, CPB=cardiopulmonary bypass (or “pump” procedure).

The group with pump procedures included a higher percentage of men, and those with a higher prevalence of coronary artery disease, prior CV intervention, number of diseased vessels, hypertension, diabetes, cardiogenic shock, and left main disease; whereas, patients with off-pump procedures were slightly older, included a greater proportion of Blacks and Hispanics, patients with Medicare/Medicaid insurance and those with urgent CABG surgery, and patients with a higher prevalence of peripheral vascular disease, renal failure, and moderate/severe lung disease (Table 1). The majority of patients in each group were taking aspirin and/or beta blockers preoperatively.

**Figure 1:**
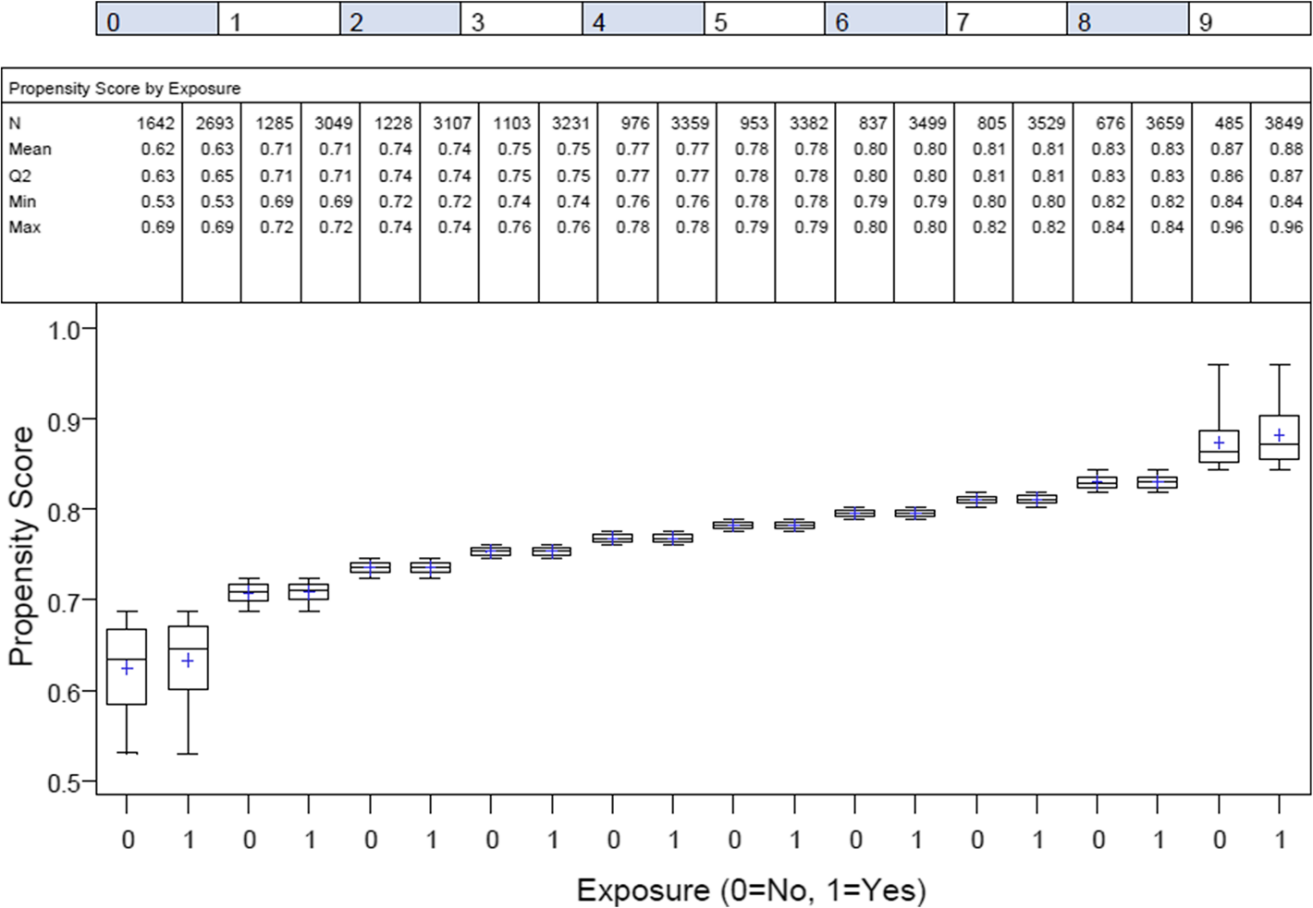
**Boxplot of propensity scores by exposure status and quantiles of propensity score distribution.** Legend. Final analytical dataset including a total of 43,347 coronary artery bypass patients. 1 and 0 designates patients with pump and off-pump exposure status, respectively.

The final propensity score model included age, race, myocardial infarction, number of diseased vessels, prior intervention, congestive heart failure, hypertension, smoking, preoperative status (ie, urgency of the procedure), lung disease, peripheral vascular disease, cerebrovascular disease, renal failure, ejection fraction, left main disease, endocarditis, and cardiogenic shock, each of which was significant in the model (p<0.01, AUC=0.679 [95% CI: 0.673, 0.685], p<.0001). There was fairly good overlap in the resulting propensity score distributions, except for the tail ends (Figure 1). The final dataset, after asymmetric trimming to improve overlap of the propensity score distributions and balance among baseline covariates, included a total of 43,347 or 90% of the original dataset.

### Cancer incidence and stage

Of the 43,347 patients included in the final analysis, a total of 2,960 (6.8%) patients were diagnosed with a total of 3,182 primary cancers (Table 2). The majority of patients diagnosed with cancer were male (79.2%), white (87.3%), and the median age at the time of cancer diagnosis was 74 yrs. Of those diagnosed with cancer, the most commonly reported cancers types included prostate (25.2%), lung and bronchus (15.0%), colorectal (14.1%), and cancer of the urinary bladder (8.2%).

**Table 2:** **Relative risk of cancer**, **including any cancer and tumor**-**specific cancers**

	**CABG patients**^**a **^**(n=43,347)**									
	**Pump**^**b **^**(n=33,357)**		**Off Pump (n=9,900)**		**Unadjusted**			**Adjusted**^**c**^		
	**n**	**%**^**d**^	**n**	**%**^**d**^	**RR**^**e**^	**(95% CI)**	**p**-**value**	**RR**^**e**^	**(95% CI)**	**p**-**value**
Patients ≥1 primary cancer^f^	2,388	7.2%	572	5.8%						
Patients w/tumor-specific cancers^g^										
• Lung and bronchus	369	1.1%	75	0.8%	1.24	(0.94-1.62)	0.125	1.36	(1.02- 1.81)	0.034
• Prostate	609	1.8%	137	1.4%	1.13	(0.85-1.51)	0.401	1.25	(0.92-1.68)	0.149
• Pancreas	63	0.2%	18	0.2%	0.86	(0.57-1.32)	0.495	0.95	(0.62-1.47)	0.820
• Stomach	65	0.2%	23	0.2%	0.73	(0.43-1.22)	0.226	0.80	(0.48-1.35)	0.404
• Breast	100	0.3%	30	0.3%	0.84	(0.52-1.35)	0.467	0.92	(0.57-1.51)	0.752
• Colon/rectum	338	1.0%	79	0.8%	1.11	(0.90-1.38)	0.343	1.22	(0.98-1.53)	0.083
• Kidney/renal pelvis	93	0.3%	25	0.3%	0.95	(0.75-1.21)	0.676	1.05	(0.83- 1.32)	0.698
• Urinary bladder	197	0.6%	45	0.5%	1.07	(0.79-1.45)	0.672	1.18	(0.84-1.66)	0.339
• Corpus uterus	31	0.1%	3	0.0%	***	*********	****	***	*********	****
• Non-hodgkin lymphoma	90	0.3%	23	0.2%	0.94	(0.65-1.35)	0.721	1.03	(0.71- 1.50)	0.874
• Skin melanoma	116	0.3%	19	0.2%	1.50	(0.99-2.27)	0.053	1.66	(1.08- 2.55)	0.022

^a^final dataset after asymmetric trimming of the propensity score model to improve overlap of the propensity score distributions for patients with and without CPB CABG surgery. The final dataset includes 43,347 patients or 90% of the original dataset.

^b^includes CABG patients with ≥1 on-pump procedure prior to incident cancer.

^c^model adjusted for age at time of surgery, gender, race, year of surgery, use of blood products, and propensity score.

^d^%=number of patients with tumor-specific cancer/ total number in group*100.

^e^risk ratio for pump/off-pump modeled using Cox proportional hazards model with a robust covariance matrix that accounted for survival times for individuals within a hospital. For patients with multiple primaries, other cancers are censored at the time of occurrence. Competing risk model with regression of exposure on cause-specific hazard.

^f^multiple primary tumor-specific cancers (e.g. prostate, colorectal) may have been reported for any given patient. For the relative risk of any cancer, the first cancer reported for patients with multiple primaries was used in the time to event analysis.

^g^for tumor-specific cancers, other cancers were censored at the time of diagnosis.

*results for cancers with cell count ≤5 were suppressed to as a way to ensure statistical reliability and protect patient confidentiality.

CABG=coronary artery bypass graf, CPB=cardiopulmonary bypass (or “pump” procedure).

The risk of developing cancer was proportionally greater in patients who underwent pump procedures compared with off-pump procedures (7.2% versus 5.8%, respectively), but the difference did not achieve statistical significance with or without adjustment for baseline risk factors (crude RR=1.06 [95% CI: 0.86-1.30, p=0.59], adjusted RR=1.17 [95% CI: 0.93-1.47, p=0.19]) (Table 2). These findings correspond to a cancer rate of 3.8 per 100,000 person-years of risk in the group with pump procedures and 3.5 per 100,000 person-years of risk in the group with off-pump procedures.

Of the 11 tumor-specific analyses performed, there was an increased risk of skin melanoma [RR=1.66 (95% CI, 1.08-2.55: p=0.02)], lung cancer [RR=1.36 (95% CI, 1.02-1.81: p=0.03)]), and a borderline increase in the relative risk of colorectal cancer (1.22 [95% CI: 0.98-1.53], p=0.08) after adjustment for baseline risk factors (Table 2). Similar findings were observed using standard Kaplan Meier methods (data not shown). For those cancers with a reported stage, there was no significant difference in the stage of cancers between the groups with and without pump exposure (p=0.65). Approximately 20% of reported cancers were metastatic at the time of diagnosis.

### Cancer-specific mortality

**Figure 2:**
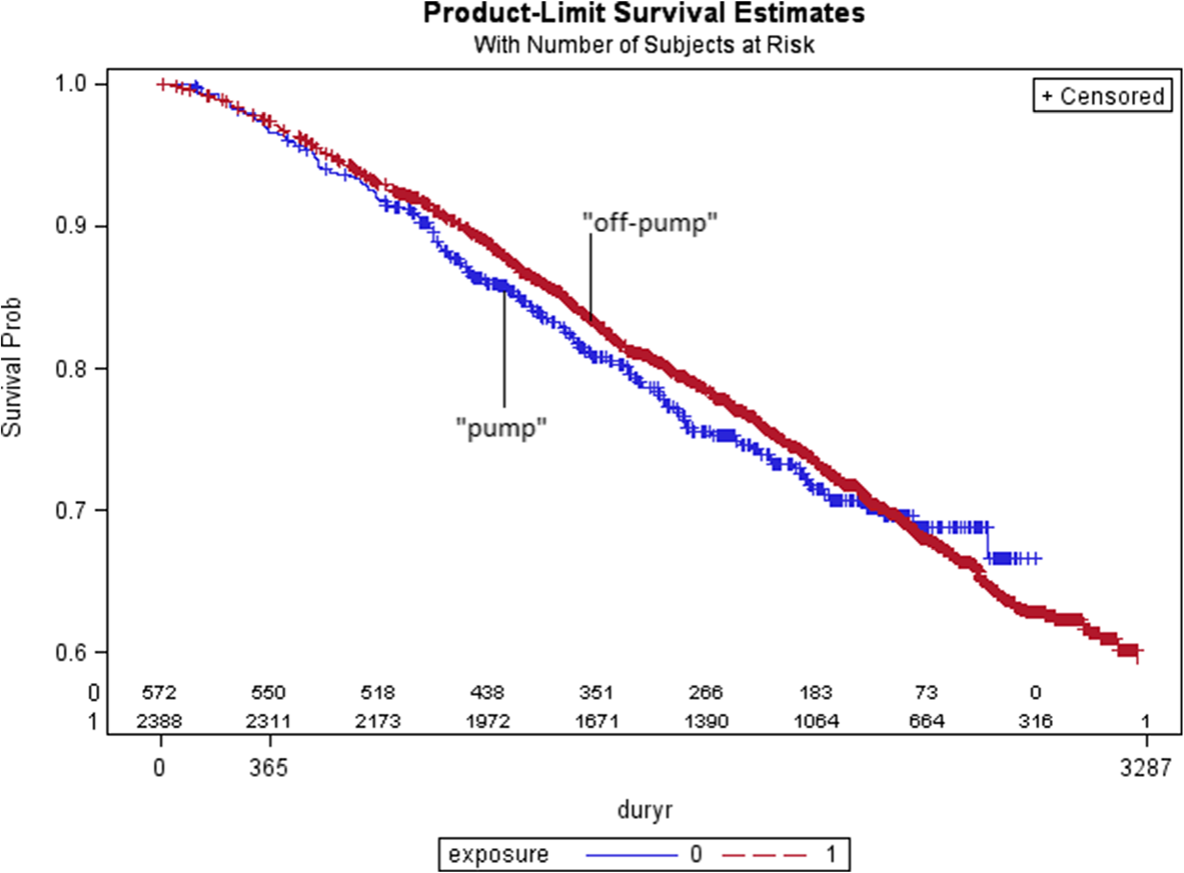
**Kaplan Meier survival curve for cancer-specific mortality for patients with open-heart surgery and cancer diagnosed during follow-up, by CPB status Legend: Includes 2,960 cancer patients in study cohort.**

Of the 43,347 patients with isolated CABG surgery, the adjusted relative risk of cancer-specific mortality was 1.16 (95% CI: 0.92-1.46, p=0.20) (Table 3). Cardiovascular and other-cause specific mortality was also increased for those who underwent pump versus off-pump surgery (1.15 (95% CI: 0.86-1.55, p=0.34) and 1.20 (95% CI: 0.65-2.19, p=0.56), respectively. Of those patients with a cancer diagnosis after surgery, the adjusted risk of cancer-specific mortality was 1.12 (95% CI: 0.89-1.41, p=0.33) (Table 4). A Kaplan Meier plot illustrating the risk of cancer-specific mortality for those patients with cancers diagnosed after surgery is presented in Figure 2. No trend was observed for cancers diagnosed within shorter timeframes after surgery (Table 4). 

The lack of temporal association between surgery and cancer diagnosis was also observed in a separate study of CPB in patients with a prior cancer diagnosis (data unpublished). In this separate study, the relative risk for patients with pump versus off-pump procedures for cancers diagnosed within a year prior to surgery was 1.05 (95% CI, 0.85-1.30: p=0.67) compared to HR=1.10 (95% CI, 0.87-1.42: p=0.41) and HR=1.07 (95% CI, 0.84-1.36: p=0.57) for those patients with cancers diagnosed 2 years and 4 years prior to surgery, respectively.

**Table 3:** **Cause**-**specific mortality for isolated open**-**heart surgery patients with no prior cancer diagnosis**, **by CPB status**

	**CABG patients**^**a **^**(n=43,347)**									
	**Pump**^**b**^		**Off pump**		**Kaplan Meier**^**c**^			**Competing risk model**^**c**^		
	**(n=33,357)**		**(n=9,990)**							
	**n**	**%**^**d**^	**n**	**%**^**d**^	**HR**^**e**^	**(95% CI)**	**p-value**	**HR**^**e**^	**(95% CI)**	**p-value**
**Cancer-specific mortality**										
• Unadjusted model	923	2.8%	218	2.2%	1.02	(0.76-1.37)	0.898	1.02	(0.76-1.37)	0.898
• Adjusted model^f^					1.13	(0.90-1.42)	0.306	1.16	(0.92-1.46)	0.203
**Cardiovascular-specific mortality**										
• Unadjusted model	2,979	8.9%	754	7.5%	1.01	(0.78-1.31)	0.938	1.01	(0.78-1.31)	0.938
• Adjusted model^f^					1.26	(0.89-1.79)	0.186	1.15	(0.86-1.55)	0.344
**Other cause-specific mortality**										
• Unadjusted model	1,862	5.6%	491	4.9%	0.94	(0.73-1.22)	0.658	0.94	(0.73-1.22)	0.658
• Adjusted model^f^					1.24	(0.67-2.29)	0.495	1.20	(0.65-2.19)	0.561

^a^final dataset after asymmetric trimming of the propensity score model to improve overlap of the propensity score distributions for patients with and without CPB CABG surgery. The final dataset includes 43,347 patients or 90% of the original dataset.

^b^includes patients with ≥1 on-pump procedure prior to incident cancer.

^c^Kaplan-Meier estimate treats failures from competing causes as censored observations; competing risk model with regression of exposure on cause-specific hazard.

^d^%=number of patients who died / total number in each group*100.

^e^hazard ratio for pump/off-pump modeled using Cox proportional hazards model with a robust covariance matrix that accounted for survival times for individuals within a hospital. Zero time for analysis was time of the open-heart surgery.

^f^model adjusted for age at time of surgery, gender, race, year of surgery, use of blood products, and propensity score.

CABG=coronary artery bypass graft, CPB=cardiopulmonary bypass (or “pump” procedure).

**Table 4:** **Cancer**-**specific mortality for patients with diagnosis during follow**-**up**, **stratified by duration between surgery and diagnosis**

	**Cancer patients**^**a **^**(n=2,960)**									
	**Pump**^**b**^		**Off pump**		**Kaplan Meier**^**c**^			**Competing risk model**^**c**^		
	**(n=2,388)**		**(n=572)**							
	**n**	**%**^**d**^	**n**	**%**^**d**^	**HR**^**e**^	**(95% CI)**	**p-value**	**HR**^**e**^	**(95% CI)**	**p-value**
**Cancers diagnosed within 1 yr of surgery**										
**Number of patients with cancer**	455		126							
**Patients who died from cancer**	155	34.1%	41	32.5%						
• Unadjusted model					1.02	(0.72-1.43)	0.932	1.02	(0.72-1.43)	0.932
• Adjusted model^f^					1.01	(0.72-1.40)	0.969	1.07	(0.82-.1.41)	0.615
• Sensitivity analysis-MCD^g^	173	38.0%	43	34.1%						
o Unadjusted model					1.08	(0.77-1.52)	0.665	1.08	(0.77-1.52)	0.665
o Adjusted model^f^					1.11	(0.83-1.45)	0.514	1.19	(0.94-1.51)	0.148
**Cancers diagnosed within 2 yrs of surgery**										
**Number of patients with cancer**	868		250							
**Patients who died from cancer**	270	31.3%	75	30.0%						
• Unadjusted model					1.00	(0.78-1.28)	0.987	1.00	(0.78-1.28)	0.987
• Adjusted model^f^					1.05	(0.76-1.47)	0.762	107	(0.79-1.45)	0.656
• Sensitivity analysis-MCD^g^	299	34.4%	78	31.2%						
o Unadjusted model					1.07	(0.83-1.36)	0.611	1.07	(0.83-1.36)	0.612
o Adjusted model^f^					1.10	(0.80-1.50)	0.558	1.15	(0.87-1.54)	0.327
**Cancers diagnosed within 4 yrs of surgery**										
**Number of patients with cancer**	1,635		448							
**Patients who died from cancer**	484	29.6%	123	27.5%						
• Unadjusted model					1.00	(0.79-1.28)	0.980	1.00	(0.80-1.28)	0.980
• Adjusted model^f^					1.02	(0.79-1.32)	0.868	109	(0.85-1.39)	0.491
• Sensitivity analysis-MCD^g^	529	32.4%	132	29.5%						
o Unadjusted model					1.02	(0.80-1.29)	0.874	1.02	(0.89-1.29)	0.874
o Adjusted model^f^					1.05	(0.80-1.38)	0.678	1.10	(0.87-1.40)	0.417
**Cancers diagnosed at any time during follow-up**										
• Unadjusted model	668	28.0%	141	24.7%	0.96	(0.78-1.17)	0.665	0.96	(0.78-1.17)	0.665
• Adjusted model^f^					1.06	(0.85-1.32)	0.595	1.12	(0.89-1.41)	0.330
• Sensitivity analysis-MCD^g^										
o Unadjusted model	730	30.6%	154	26.9%	0.96	(0.78-1.16)	0.648	0.96	(0.78-1.16)	0.648
o Adjusted model^f^					1.04	(0.98-1.09)	0.211	1.11	(0.89-1.39)	0.366

^a^final dataset after asymmetric trimming of the propensity score model to improve overlap of the propensity score distributions for patients with and without CPB CABG surgery. The final dataset includes 43,347 patients or 90% of the original dataset.

^b^includes patients with ≥1 on-pump procedure prior to incident cancer.

^c^Kaplan-Meier estimate treats failures from competing causes as censored observations; competing risk model with regression of exposure on cause-specific hazard.

^d^%=number of patients who died / total number in each group*100.

^e^hazard ratio for pump/off-pump modeled using Cox proportional hazards model with a robust covariance matrix that accounted for survival times for individuals within a hospital. Zero time for analysis was time of the open-heart surgery.

^f^model adjusted age at time of initial cancer diagnosis, gender, race, cancer type, cancer stage, cancer treatment (e.g. chemotherapy, surgery), duration between surgery and cancer diagnosis, use of blood products, year of surgery, and propensity score.

^g^the analysis used cancer-specific mortality reported as primary cause of death or underlying cause of death using data from the NCHS multiple cause of death file.

CABG=coronary artery bypass graft, “pump” procedure= CPB=cardiopulmonary bypass.

Although the results of each analysis presented herein demonstrates an increased mortality risk for those with CPB exposure, none of the results reached statistical significance. Similar findings were observed using standard Kaplan Meier methods and using the NJSCR multiple cause of death file (Tables 3, 4). For each analysis of cancer-specific mortality, the proportional hazards assumption was satisfied.

## Discussion

This is the first population-based multicenter cohort study in patients with underlying cardiovascular disease and no pre-existing cancer diagnosis which used robust propensity score modeling to adjust for baseline imbalances and which accounted for competing risks. Results of this research show a statistically significant increase in the relative risk of skin melanoma (RR=1.66: 95% CI, 1.08-2.55: p=0.02), cancer of the lung and bronchus (RR=1.36: 95% CI, 1.02-1.81: p=0.03), and an increase in overall cancer incidence in patients who underwent isolated CABG surgery with cardiopulmonary bypass compared to those patients undergoing off-pump surgery (RR=1.17: 95% CI, 0.93-1.47: p=0.19). The largest increase in cancer incidence, was observed for skin melanomas. This is consistent with the underlying hypothesis that immunosuppression and decreased immunosurveillance are risk factors for melanoma. Prior studies have shown an increased risk of skin melanoma with chronic immune suppression [18]. The possible etiology of the observed association of CPB with lung cancer development is less clear. Although prior studies have shown an increased risk of lung cancer in patients with HIV and heart transplant recipients, it is unclear if these prior findings are related to immune suppression, medical surveillance bias, or an increase in behavioral risk factors such as smoking for which there may be residual confounding [19,20]. In the present study, there was no notable difference in the history of smoking between the pump versus off-pump groups.

Compared to open-heart surgery patients undergoing off-pump procedures, the results also show a non-statistically significant increase in the risk of cancer-specific mortality for patients who underwent cardiopulmonary bypass surgery prior to any diagnosis of cancer 1.16 (95% CI, 0.92-1.46: p=0.20) as well as a non-statistically significant increase in the case-fatality rate for those cancer patients who underwent surgery with cardiopulmonary bypass prior to the cancer diagnosis (1.12: 95% CI, 0.89-1.41: p=0.33). No difference was observed in cancer stage at the time of diagnosis (p=0.65). The time between surgery and cancer diagnosis, which would serve as the most compelling evidence given its temporal association, did not show any impact on the relative risk of mortality. Even so, there were too few events to draw any definitive conclusions.

The general strengths of this study includes its population-based cohort design, the use of medical information recorded by clinicians rather than self-reports by patients or by proxy, the virtually complete accounting of open-heart surgeries given the need for hospitalization of these patients, and the mandatory reporting requirements for cancers in the State of New Jersey. The number of patients with procedures or relocation outside of New Jersey is assumed to be minimal and non-differential for patients with and without cardiopulmonary bypass surgery. Other strengths of this study include the completeness of vital status records captured using a variety of methods including, but not limited to, a review of state and national death files, state taxation files, hospital discharge files, Medicare and Medicaid files, and motor vehicle registration records, and the ability to perform sensitivity analyses using the National Center for Health Statistics (NCHS) Multiple Cause of Death file that captures a cancer diagnosis reported as the primary or underlying cause of mortality on the death certificate. Additionally, robust propensity score modeling was used to help minimize potential confounding, and competing risk methods were used given that competing risks are of notable concern due to the age and prevalence of comorbidities in this particular patient population.

Although this research entailed one of the most robust assessments of cancer incidence and cancer-specific mortality in CPB patients to date in a population-based setting, there are design limitations. First, although robust probabilistic record linkage methods were used to link records in the different state-wide databases, there is still the potential for selection bias and information bias resulting from this process. As well, the generalizability of this study is limited given asymmetric trimming of ~10% of the original database to improve overlap in propensity score distributions. Additionally, even with the use of propensity score adjustment for important risk factors, there is still the chance of imbalances between the groups in important unmeasured confounders and residual confounding due to lack of detailed information collected for other important risk factors included in the propensity score modeling. Lastly, with no control for false discovery rates and multiple testing, spurious results are possible and, as such, the results should be interpreted with caution.

## Conclusion

The data suggest there may be some degree of association between CPB and cancer progression. However, if real, the effect is likely to be modest at best. Although clinical practice guidelines will not likely change based on these findings, the results may assure clinicians that the choice of cardiopulmonary bypass should be determined by other clinical considerations. Further research may still be warranted to assess whether the transient immunosuppression associated with CPB can promote the spread and growth of pre-existing cancer cells with particular focus on skin melanoma and lung cancer which had the largest association in this study.

## Competing interests

The authors declare that they have no competing interests.

## Authors’ contributions

CAP, SM, DA, BH, and KD have each made substantial contributions to the study design, analysis, interpretation of the data, and drafting of the manuscript. JK assured the creation and maintenance of the longitudinal information of MIDAS, and made contributions to the study design. All authors have reviewed the manuscript critically for important intellectual content and given final approval of the version to be published.

## Authors’ informations

CAP is a lead epidemiologist for a major pharmaceutical company, specializing in cardiovascular disease, diabetes, and urology, and is a member of the international society of pharmacoepidemiology (ISPE) and international society of pharmacoeconomics and outcomes research (ISPOR). SM is a clinical epidemiologist, a general internist and pediatrician with an interest in the metabolic syndrome and cancer. He currently is a consultant in global health outcomes research. DAA is the Chief of Surgical Oncology at The Rutgers Cancer Institute of New Jersey and Robert Wood Johnson Medical School. His clinical practice as a cancer surgeon led him to become interested in the role of immunosuppression in cancer pathogenesis. BH is a biostatistician with many years’ experience conducting epidemiological studies. He has a special interest in survival models and hazards regression. JK is the Director of the Cardiovascular Institute, Associate Dean for Cardiovascular Research at Rutgers-Robert Wood Johnson Medical, and Principal Investigator for MIDAS. KD is the Chair of Epidemiology at the Rutgers School of Public Health and a member of the Cancer Institute of New Jersey. He is a cancer epidemiologist with a research focus in understanding cancer progression.

## Pre-publication history

The pre-publication history for this paper can be accessed here:

http://www.biomedcentral.com/1471-2407/13/519/prepub
